# Overexpression of DNA damage-induced 45 α gene contributes to esophageal squamous cell cancer by promoter hypomethylation

**DOI:** 10.1186/1756-9966-31-11

**Published:** 2012-02-08

**Authors:** Bao xiang Wang, Bang Liang Yin, Bin He, Chen Chen, Ming Zhao, Wei xing Zhang, Zhen Kun Xia, Yi zhi Pan, Jing qun Tang, Xin min Zhou, Ni Yin

**Affiliations:** 1 Department of cardiothoracic Surgery, Second Xiangya Hospital of Central South University, Changsha, Hunan, PR China; 2 Hunan Key Laboratory of Medical Epigenomics, Department of Dermatology, Second Xiangya Hospital of Central South University, Changsha, Hunan, PR China; 3 Department of cardiothoracic Surgery, Xiangya Hospital of Central South University, Changsha, Hunan, PR China; 4 Department of cardiothoracic Surgery, Third Xiangya Hospital of Central South University, Changsha, Hunan, PR China

**Keywords:** Esophageal squamous cell cancer, GADD45α, DNA methylation, DNA damage

## Abstract

**Background:**

Environmental factors-induced dysfunction of esophageal squamous epithelium, including genomic DNA impairment and apoptosis, play an important role in the pathogenesis of esophageal squamous cell cancer. DNA damage-induced 45α (GADD45α) has been found promoting DNA repair and removing methylation marker, Therefore, in this study we will investigate whether GADD45α expression is induced and its mechanism in esophageal squamous cell cancer.

**Methods:**

Two human esophageal squamous cell lines (ESCC), ECA109 and KYSE510 were cultured in RPMI-1640 medium supplemented with 10% fetal bovine serum (FBS). Lipofectamine 2000 was used to transfect cells. mRNA level of GADD45α was measured by reverse transcription-quantitive PCR (RT-qPCR), protein level of GADD45α was detected by western blot and Immunohistochemistry. Global DNA methylation of tissue sample was measured using the Methylamp Global DNA Methylation Quantification Ultra kit (Epigentek Group) and promoter methylation was measured by bisulfite sequencing.

**Results:**

GADD45a mRNA and protein levels were increased significantly in tumor tissue than that in adjacent normal tissue. Hypomethylation of global genomic DNA and GADD45α promoter were found in ESCC. The cell sensitivity to Cisplatin DDP was decreased significantly in Eca109 and Kyse510 cells, in which GADD45α expression was down-regulated by RNA interference (RNAi). In addition, silence of GADD45a expression in ESCC cells inhibited proliferation and promoted apoptosis.

**Conclusion:**

Overexpression of GADD45α gene is due to DNA hypomethylation in ESCC. GADD45α may be a protective factor in DDP chemotherapy for esophageal squamous cell carcinoma.

## Background

Esophageal cancer is the eighth most common malignancy and the sixth most common cause of cancer-related death worldwide [1,2], its prevalence and death rate are continuously increasing and thus has become a major health concern[3]. Esophageal squamous cell carcinoma (ESCC) is the predominant type of esophageal cancer, comprising almost 95% of cases. The development of ESCC is strongly correlated with a number of dietary and environmental factors, such as alcohol consumption, smoking, hot food, pungent meal and high levels of nitrates in the soil and drinking water [4]. These pathogenic factors may destroy esophageal squamous epithelium, thus epithelial cells suffer from DNA damage and apoptosis [5], which may result in genomic instability and cell transformation. Although multiple genetic and epigenetic changes have been reported in ESCC development and progression [6-15], the precise molecular mechanisms still remain unclear.

Growth arrest and DNA damage-induced 45α (GADD45α), a nuclear protein, belongs to the DNA damage-induced 45 family, has been considered to participate in cellular response to a variety of DNA damage agents. GADD45α-null mice generated by gene targeting exhibits severe genomic instabilities [16]. Most strikingly, mice lacking the GADD45α gene are susceptible to DNA damage-induced tumors, including carcinogenesis induced by ionizing radiation, UV radiation and dimethylbenzanthracene (DMBA) [17,18]. A recent study showed that GADD45α has a key role in active DNA demethylation and its overexpression activates methylation-silenced reporter plasmids and promotes global DNA demethylation. [19]

DNA methylation in cancer tissue was first observed more than two decades ago[20] and may be linked to carcinogenesis[21].The in-depth investigations of the relationship between DNA methylation and gene expression provide very important information for understanding cancer and finding the effective therapy strategy. Presently, GADD45α expression and promoter methylation status in ESCC have not been reported. We hypothesized that epigenetic regulation on GADD45α may play an important role in ESCC development.

In this study, we first detected GADD45α mRNA expression by reverse transcription- quantitative polymerase chain reaction (RT-qPCR) and DNA methylation status by bisulfite sequence in 40 primary ESCC tissues and corresponding normal tissues. We further evaluated the correlations among GADD45α mRNA, DNA methylation and the tumor clinical pathologic stages. RNAi was subsequently applied to investigate the role of GADD45α on cell proliferation and apoptosis in esophageal squamous cancer cell line.

## Methods

### Samples

Tumor tissue and adjacent normal tissue(confirmed by hematoxylin and eosin) were obtained from 40 esophageal squamous cell cancer patients(Table 1) who underwent curative resection at the second Xiang Ya Hospital (Hunan, China) between June 2010 and January 2011 after informed consent was obtained from all the patients. None of the patients received chemotherapy and radiotherapy before surgery. Tissues were fixed in 10% buffered formaldehyde solution for pathological diagnosis and imunohistochemical staining and frozen in liquid nitrogen for DNA, RNA and protein isolation.

**Table 1:** Clinical character of ESCC patients

Patient/age/sex	T/N/M/G	Condition of one year after operation
1/64/M	T3/N1b/M0/G1-G2	alive

2/68/M	T3/N0/M0/G1	alive

3/58/M	T3/N0/M0/G1	alive

4/61/M	T0/N0/M0/G1	alive

5/63/M	T1b/N0/M0/G2	alive

6/76/F	T2/N0/M0/G3	alive

7/62/M	T3/N1a/M0/G1	alive

8/55/M	T3/N1a/M0/G2	alive

9/63/M	T2/N1a/M0/G2	alive

10/47/M	T2/N1a/M0/G1	alive

11/42/M	T2/N0/M0/G2	alive

12/55/M	T3/N0/M0/G1	alive

13/56/M	T1b/N0/M0/G1-G2	alive

14/56/M	T3/N1a/M0/G1	dead

15/73/M	T4/N0/M1/G2-G3	alive

16/61/M	T3/N0/M0/G2	alive

17/67/M	T2/N0/M0/G2	alive

18/59/M	T2/N1a/M0/G2	alive

19/55/M	T3/N1b/M0/G2	alive

20/58/M	T3/N1a/M0/G2	alive

21/57/M	T2/N1a/M0/G2	alive

22/65/M	T2/N1a/M0/G2-G3	metastasis in lymph node of nest

23/54/M	T3/N1a/M0/G2	alive

24/56/M	T3/N0/M0/G2	alive

25/74/M	T2/N1a/M0/G2	metastasis in lymph node of nest

26/51/M	T2/N1b/M0/G1	alive

27/52/M	T3/N1a/M0/G1	alive

28/60/M	T2/N1a/M0/G2	alive

29/47/M	T2/N0/M0/G2	alive

30/55/M	T3/N1b/M0/G2	alive

31/68/M	T3/N0/M0/G2	alive

32/71/M	T3/N1a/M0/G2	alive

33/55/M	T2/N0/M0/G1	alive

34/69/M	T2/N0/M0/G2	alive

35/55/M	T2/N1b/M0/G2	alive

36/59/M	T2/N1b/M0/G1-G2	metastasis in liver

37/60/M	T2/N0/M0/G2	alive

38/54/M	T2/N1a/M0/G2	alive

39/62/M	T3/N1b/M0/G2	alive

40/59/M	T1b/N0/M0/G1-G2	alive

### Cell lines

Two human esophageal squamous cell lines, ECA109 and KYSE510 were obtained from American Type Culture Collection (ATCC) and Deutsche Sammlung von Mikroorganismen und Zellkulturen GmbH (German Collection of Microorganisms and Cell Cultures) (DSMZ) respectively, and were grown in Hyclone RPMI-1640 medium (Thermo scientific, Beijing, China) supplemented with 10% fetal bovine serum (FBS), 100 U/ml of penicillin sodium, and 100 mg/ml of streptomycin sulfate, cultured at 37°C in humidified air containing 5% carbon dioxide air atmosphere. HEEpiC(Human esophageal epithelial cells) cell line was obtained from San Diego, US (ScienCell). And they were cultured and proliferated in Epithelila Cell Medium-2 at 37°C in humidified air containing 5% carbon dioxide air atmosphere.

### Real-time reverse transcription-polymerase chain reaction (RT-PCR)

Total RNA was isolated from tumor and adjacent normal tissue using Trizol reagent according to standard protocol (Invitrogen, USA). cDNA synthesis was performed using RevertAid™ First Strand cDNA Synthesis Kit (Fermentas, Burlington, Canada) and 1 μg of total input RNA according to the manufacturer's instructions. Real-time quantitative PCR was performed using a Rotor-Gene3000 (Corbett Research, NSW, Australia) and mRNA levels were quantified using SYBR Premix Ex TaqTM real-time PCR Kit (TaKaRa Biotech [Dalian] Co., China). β-actin was also amplified and used as a loading control. The primers for GADD45α, GADD45β, GADD45γ, and β-actin used were shown in Table 2.

**Table 2:** Primers of genes

Gene	primers
GADD45α,	PF:5'-GCCTGTGAGTGAGTGCAGAA-3',

	RF: 5'-CCCCACCTTATCCATCCTTT-3'

GADD45β	PF:5'-TCGGCCAAGTTGATGAATG-3':

	RF: 5'-CAGAAGGACTGGATGAGCGT-3'

GADD45γ	PF:5'-CGTCTACGAGTCAGCCAAAG-3'

	RP:5'-GCCTGGATCAGCGTAAAAT-3'

β-ACTIN	PF:5'GCACCACACCTTCTACAATGAGC'3

	RP:5'GGATAGCACAGCCTGGATAGCAAC'3

### Bisulfite genomic sequencing

Bisulfite conversion was performed using the Epitect Bisulfite kit (Qiagen Germany) according to the manufacture's protocol. The 484 bp GADD45α promoter fragments were amplified using nested PCR, and then cloned into a pGEM-T vector (Promega USA). The 5 independent clones were then sequenced for each of the amplified fragments. The primers for GADD45α were as follows: first round, forward 5'-TGTGGGCTGTGTGGGTGTCAGATGG-3' and reverse 5'-GAGGGTTGGCAGGATAACCCC-3'; the second round, forward 5'-AAAGTTTTTTATTTTTAATGGTTTTT-3' and reverse 5'-GGTTAAATTGTTGGAGTAGGTTGAT-3 '.

### Global DNA methylation detection

Genomic DNA was isolated from tissue of tumor and normal adjacent using the TIANamp Genomic DNA kit (Tiangen Biotech). Global methylation levels were measured using the Methylamp Global DNA Methylation Quantification Ultra kit (Epigentek Group) according to the manufacturer's instruction. In this assay, DNA is immobilized to wells specifically coated with a specific DNA affinity substratum. The methylated fraction of DNA can be recognized with a 5-methylcytosine antibody and quantified through an ELISA-like reaction. Absorbance was measured at 450 nm.

### Immunohistochemistry

The paraffin sections were made from the tumor tissue and adjacent normal tissue of patients. All the paraffin sections were 4 um thick. Firstly the paraffin sections were baked at 60°C for 1 h and were dew axed with turpentine Oil and 100%, 95%, 75% and 50% alcohol one by one. The sections were incubated in 1.2% hydrogen peroxide for 10 min and rinsed in phosphate balanced solution(PBS), pH 7.4, for 12 min. The sections were then blocked for 1 h with normal goat serum. After incubating with the primary rabbit anti-human antibody for 1 h at room temperature, the cryostat sections were washed in PBS and incubated with a secondary anti-rabbit biotinylated antibody for 30 min, and subsequently with the streptavidin-HRP complex for 10 min, rinsed in PBS. And then the sections were stained with ACE solution for 10 min. Finally the sections were stained with haematoxylin. The results were analyzed with Point rating method. We used the percentage of GADD45α-positiv stained cells and the intensity of GADD45α expression by the tumor cells to grade all the samples. And the multiplication of these two grading scores calculates the immunoreactive score for GADD45α expression (GADD45α-IRS) in stained tissue (%GADD45α -positive tumor cells × staining intensity = GADD45α-IRS).

### Western blot analysis

For tumor and adjacent normal tissues were frozen in liquid nitrogen and powdered with mortar and pestle and lysed by cell lysis buffer. Samples were transferred to microcentrifuge tubes, homogenized, and protein pelleted by microcentrifugation at 14 000 rpm and 4°C for 15 min. The samples were diluted with 2 × sodium dodecyl sulfate (SDS) sample buffer and boiled. SDS samples were resolved by polyacrylamide gel electrophoresis and transferred onto polyvinylidene difluoride membrane. The membranes were incubated with the primary antibodies and then with horseradish peroxidase-conjugated secondary antibodies. The immunoblotted proteins were photographed using Lumiglo Reagent (#7003, CellSignaling Inc.).

### Transfections

Control small interfering RNA (siRNA) and siRNA targeting GADD45α were designed and synthesized at Qiagen USA. The sequences of the siRNA for GADD45α were as follows: target sequence 5'-AACATCCTGCGCGTCAGCAAC-3', sense strand5'-CAUCCUGCGCGUCAGCAACTT-3', Antisense strand: 5'-GUUGCUGACGCGCAGGAUGTT-3'. Lipofectamine 2000 was used to transfect siRNA and negative control into the two cell lines ECA109 and kyse510.

Total RNA was extracted from esophageal squamous cell cancer tissue, and GADD45α cDNA was amplified by RT-PCR. The PCR product was doubly digested by Xbal and Sall, and then recombined into eukaryotic expression vector. Then, pIRES-GFP-GADD45α was obtained by G418 selection, and then pIRES-GFP- GADD45α and pIRES-GFP were transfected into human esophageal squamous epithelial cells with lipidosome-packaged method. Meanwhile, the transfected cells were selected by G418, and then stable transfected cell lines were obtained.

### Drug sensitivity assay

Cells (1 × 10^5^/ml) were cultured in 96 cell plates after 1 day of transfectioin. After 1 day of culturing, the cells were treated with various concentrations of cisplatin (DDP). After 24 h, 48 h and 72 h of treatment, 20 ul MTT (Roche, Mannheim Germany) solution (2 mg/ml) was added to each well, and the plate was then incubated at 37°C for 4 h. Absorbance in individual wells was determined at 570 nm using a micro plate reader (Model 450, Bio-rad, CA). Three independent experiments were carried out for each treatment.

### Flow cytometric analysis

Eca109 and Kyse510 (4 × 10^5^) were seed in 12-well plates and then were transfected. Transfected Cells were harvested at 24 h, 48 h, and 72 h for flow cytometric analysis. Cells were washed twice with PBS and then incubated with 20 ug/ml PI, 100 ug/ml RNase, and 0.1% triton X-100 in PBS for 30 min in the dark. The PI stained cells were analyzed for cell cycle distribution and apoptosis by using a FACScalibur instrument (BD bioscience, San Jose, A) equipped with Cell Quest software (Becton Dickinson).

### Statistical analysis

Students's *t*-test for equality of means was used to compare values. Person's correlation coefficient was used to determine the relationship. *P *values less than 0.05 were considered significant. All analyses were performed with SPSS version 16.0 software.

## Results

### Overexpression of GADD45α in tumor tissue of ESCC

**Figure 1:**
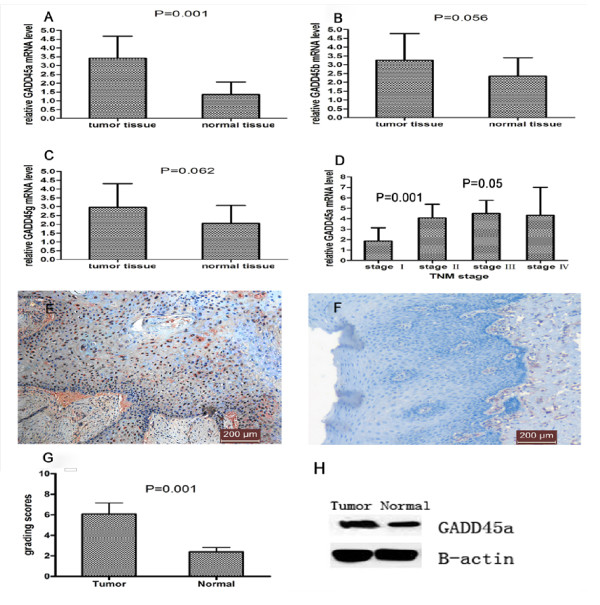
**Growth arrest and DNA damage-induced 45a (GADD45α), GADD45β, GADD45γ gene expression in tumor tissue compared with adjacent normal tissue from the same esophageal squamous cancer patients**. A, B and C, Relative expression of GADD45a, GADD45β, GADD45γ mRNA in tumor tissues from ESCC patients was measured by quantitative real-time PCR. Results were normalized to the level of β-actin (loading control). D shows the different expression levels of GADD45α in various TNM stages. G. Protein levels of GADD45α in tumor tissue and adjacent normal tissue from ESCC patients were assessed by immunohistochemistry. E shows the representative GADD45α-positive staining in tumor tissue from ESCC patients. GADD45α protein is mainly located in nucleolus of tumor cells. F. Negative control with less GADD45α staining in normal tissue. H Protein levels of GADD45α in tumor tissue and adjacent normal tissue from ESCC patients were assessed by western blotting.

The mRNA expression levels of GADD45α, GADD45β, GADD45γ in tumor tissue and adjacent normal tissue from ESCC were detected. GADD45α mRNA level was higher in tumor tissue than in adjacent normal tissue (*P *= 0.001) (Figure 1A and Table 3). No significant difference was found in GADD45β(Figure 1B and Table 3) and GADD45γ(Figure 1C and Table 3)mRNA levels between tumor and adjacent normal tissue. The overexpression of GADD45α in tumor tissue of ESCC was confirmed at the protein level using immunohistochemistry (Figure 1E,F and 1G) and western blotting (Figure 1H). GADD45α-positive staining was mainly located in nucleolus of tumor cells with few positive staining in surrounding matrix. To show the statistical discrimination clearly, samples with nuclear GADD45α-IRS < 5 were classified as GADD45α -negative (Figure 1F), and those with GADD45α-IRS > 5 were classified as GADD45α positive (Figure 1E), the ratio of GADD45α positive was higher in tumor tissues than normal tissues (Table 4).

**Table 3:** Relative mRNA level

Gene	tumor	normal tissue	*P *value
GADD45α	3.4315 ± 1.2301	1.3524 ± 0.7102	0.001

GADD45β	3.2564 ± 1.5201	2.3472 ± 1.0526	0.056

GADD45γ	2.9562 ± 1.3458	2.0561 ± 1.0210	0.062

**Table 4:** The result of immunohistochemistry

Tissue	GADD45α-IRS > 5	GADD45α-IRS < 5
Tumor	18/20	2/20

Normal	0/20	20/20

### The correlation between GADD45α mRNA and clinical pathological stages

We evaluated the correlation between GADD45α mRNA expressions in the ESCC tissues with clinical pathological stages. We found that the relative GADD45a mRNA level was 1.8672 ± 1.26732 in ESCC tissues from clinical stages I. Moreover, in tissues from stages II, III and IV, the relative GADD45a mRNA levels were 4.0800 ± 1.30220,4.4936 ± 1.25856 and 4.3292 ± 2.69446 respectively. (Table 5 and Figure 1D). The presence of lymph node metastasis, and poor differentiation were associated with mRNA expression levels of GADD45a in ESCC *(P *= 0.007, *P *= 0.006, *P *= 0.010 and *P *= 0.005, respectively Table 6).

**Table 5:** Correlation between the expression level of GADD45α mRNA and pTNM staging

TNM stage	Relative GADD45a mRNA	*P *value		
I	1.8672 ± 1.26732	0.026 ^a^	0.031^b^	0.029^c^

II	4.0800 ± 1.30220	0.082 ^d^	0.091^e^	

III	4.4936 ± 1.25856	0.90 ^f^		

IV	4.3292 ± 2.69446			

^a ^was the result of compare between stage I and II. ^b ^was the result of compare between stage Iand III.^c ^was the result of compare between stage Iand IV. ^d ^was the result of compare between stage IIand III. ^e ^was the result of compare between stage II and IV. f was the result of compare between stage III and IV

**Table 6:** Correlation between the expression level of GADD45α mRNA and clinic pathological factors

	Total	Relative GADD45a mRNA	*P*
Depth of invasion			

T1/2	23	2.1683 ± 1.06534	0.007

T3/4	17	4.0265 ± 1.20145	

Lymph node metastasis			

N0	18	1.5682 ± 0.76238	0.006 a

N1	14	3.8326 ± 1.25123	0.010 b

N2/N3	8	4.8352 ± 1.81245.	0.005 c

^a ^was the result of compare between N0 and N1. ^b ^was the result of compare betweenN1 and N2/N3

^c ^was the result of compare between stage N0 and N2/N3

### Hypomethylation in promoter of GADD45α in ESCC

**Figure 2:**
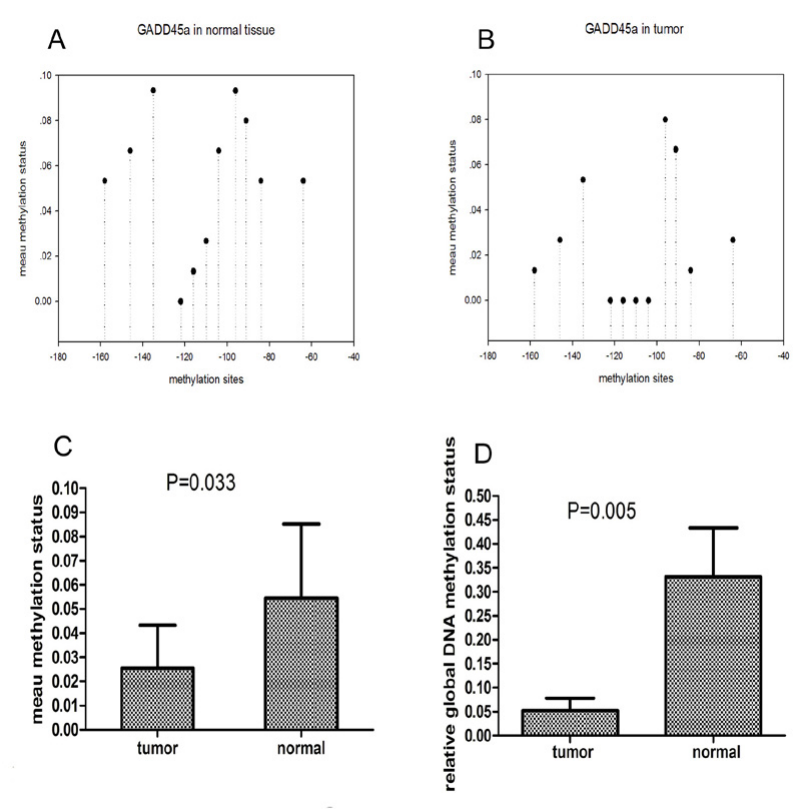
**A and B show the mean methylation status of each CG pairs in the promoter region upstream of GADD45α gene in tumor tissue and adjacent normal tissue**. Compared with adjacent normal tissue, the promoter region with 11 CG pairs (-158,-146,-135,-122,-116,-110,-104,-96,-91,-84, and-64 bp) upstream of GADD45α gene were hypomethylation in tumor tissue. C, the average methylation levels of CpG pairs in the region (-190 to-10 bp) of GADD45α promoter is significantly decreased in tumor tissue compared to the adjacent normal tissue. D shows the global DNA methylation levels of tumor and adjacent normal tissue. Compared with adjacent normal tissue, the global DNA methylation level in tumor tissue is lower.

We detected the methylation status of CG pairs in 181 bp (position-190 to -165) of GADD45α gene. Amplified fragments were cloned and five clones were sequenced for each amplification product from each subject. Figure 2 A and B show the average methylation of each 11 CG pairs within the promoter region. The mean methylation status of most CG pairs was decreased in the tumor group; there were statistically significant difference in the overall combined mean methylation status between two groups (0.0545 ± 0.03067 vs 0.0255 ± 0.01788, *P *= 0.000). (Figure 2C).

### Global DNA hypomethylation in ESCC and its correlation with clinical pathological stages

We compared the level of global DNA methylation in tumor with normal adjacent tissue. And it was found that the global DNA methylation level was significantly lower in tumor than normal adjacent tissue (Figure 2D). By evaluating the correlation between global DNA methylation level in the ESCC tissues and clinical pathological stages. We found global DNA methylation levels were higher in stages I and II than that in III and IV stages. And the same correlation was found between global DNA methylation and lymph node metastasis. A significant correlation between global DNA methylation level and clinical pathological stages was observed (*P *< 0.05) (Table 7).

**Table 7:** Correlation between the relative global DNA methylation and clinic pathological factors

	Total	Relative global DNA methylation	*P*
Depth of invasion			

T1/2	23	0.5612 ± 0.0238	0.017

T3/4	17	0.2535 ± 0.0176	

Lymph node metastasis			

N0	18	0.5852 ± 0.0185.	0.026 a

N1	14	0.3536 ± 0.0152	0.018 b

N2/N3	8	0.1568 ± 0.0123	0.006 c

^a ^was the result of compare between N0 and N1. ^b ^was the result of compare betweenN1 and N2/N3

^c ^was the result of compare between stage N0 and N2/N3

### GADD45a-siRNA transfection decreased the expression of GADD45a mRNA and protein

**Figure 3:**
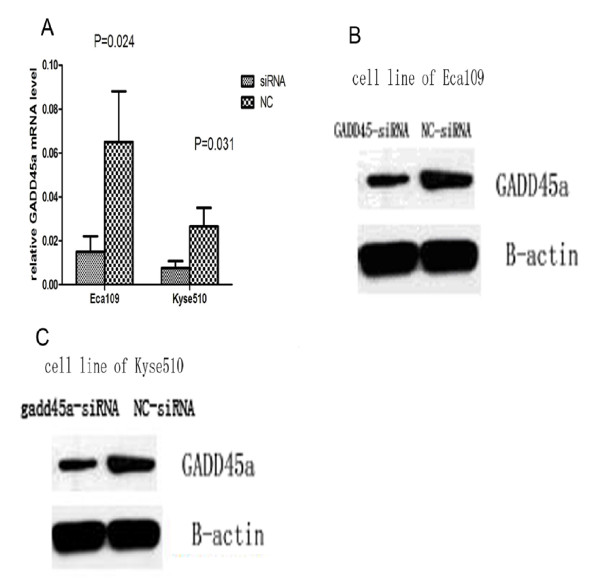
**mRNA and protein levels of GADD45α were detected by real-time PCR and western blot in ECA109 and KYSE510 with siRNA-GADD45α transfection**. A,B and C show mRNA and protein expression was inhibited significantly in ECA109 and KYSE510 transfected with siRNA-GADD45α compared with negative control.

The levels of GADD45α mRNA and protein were detected at 48 h after transfection by RT-qPCR and western blot. The levels of GADD45α mRNA and protein were decreased significantly in GADD45α knocking-down group (Figure 3A,B,C).

### Depletion of GADD45a in ESCC cells inhibited proliferation and promoted apoptosis

**Figure 4:**
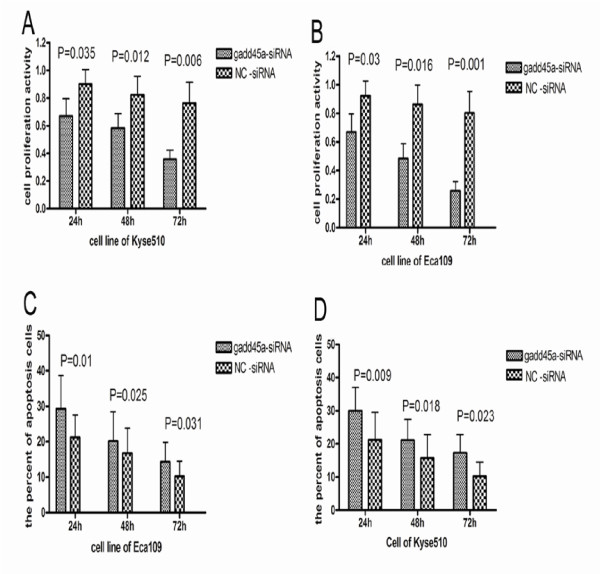
**A and B show that the ability of cell proliferation in GADD45α-siRNA group was decreased compared with NC-siRNA cells group**. C and D show the percentage of apoptotic cells in GADD45α-siRNA group and NC-siRNA group. Results confirmed that cells of apoptosis were increased significantly in the group of siRNA -GADD45α than in the group of NC-siRNA.

We observed the proliferation and apoptosis of Eca109 and Kyse510 at 24 h, 48 h and 72 h after transfection. And we found that cell proliferation of ESCC cells with GADD45α-siRNA were decreased (Figure 4A and B and Table 8) significantly. In contrast, the percentage of apoptosis cells was increased in ESCC cells with GADD45α-siRNA than negative control (Figure 4C and 4D and Table 9).

**Table 8:** The ratio of cells in S period

	GADD45s-siRNA			NC-siRNA		
	**24 h**	**48 h**	**72 h**	**24 h**	**48 h**	**72 h**

Eca109	47.84 ± 14.30	32.25 ± 11.27	25.00 ± 12.01	51.11 ± 16.00	42.50 ± 14.00	31.05 ± 13.25

Kyse510	36.63 ± 8.04	30.00 ± 13.32	20.00 ± 6.00	47.90 ± 15.34	43.50 ± 2.94	26.00 ± 6.12

**Table 9:** The percent of cell in apoptosis

GADD45s-siRNA				NC-siRNA		
	**24 h**	**48 h**	**72 h**	**24 h**	**48 h**	**72 h**

Eca109	27.33 ± 12.11	19.00 ± 2.49	9.00 ± 2.10	20.50 ± 8.83	13.41 ± 7.81	7.00 ± 4.01

Kyse510	36.63 ± 8.04	30.00 ± 13.32	20.00 ± 6.00	47.90 ± 15.34	43.50 ± 2.94	26.00 ± 6.12

### Decreased GADD45α expression by gene silence down regulated the sensitivity of Eca109 and Kyse510 cells to DDP

**Figure 5:**
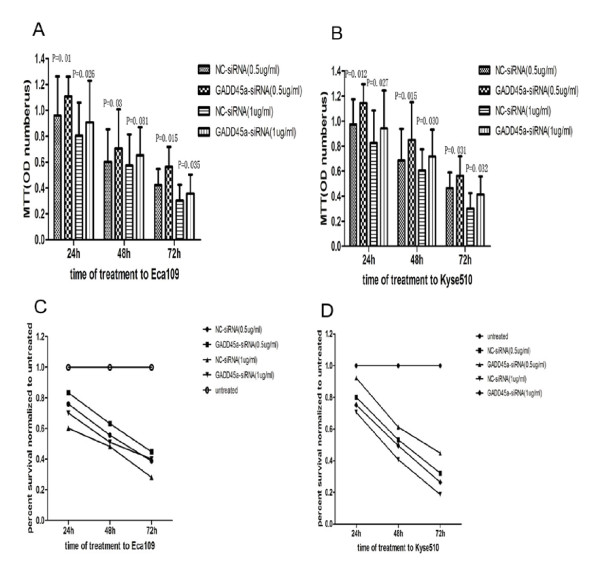
**A and B show the drug sensitivity of ECA109 and KYSE510 after transfection with siRNA-GADD45α**. ECA109 and KYSE510 cells in NC-siRNA group were more sensitive to DDP than that in two GADD45α-siRNA groups at 24 h, 48 h and 72 h with DDP treatment. Moreover, the percent of survival cells was measured by MTT value. C and D, show that the percent of survival cells at 24 h, 48 h and 72 h with DDP treatment were degraded in two GADD45α-siRNA groups compared to NC-siRNA groups.

We detected the sensitivity of Eca109 and Kyse510 cells transfected with GADD45α-siRNA to Cisplatin (DDP) at 24 h, 48 h and 72 h after treatment with DDP, at different concentration (0.5 ug/ml and 1 ug/ml)[22]. As shown in Figure 5, we observed a decreased sensitivity of Eca109 and Kyse510 cells to DDP dependent of time and dose of GADD45α-siRNA transfection in the group with knock-down GADD45α (Figure 5A,B,C,D).

### The relation of GADD45a and global DNA methylation

**Figure 6:**
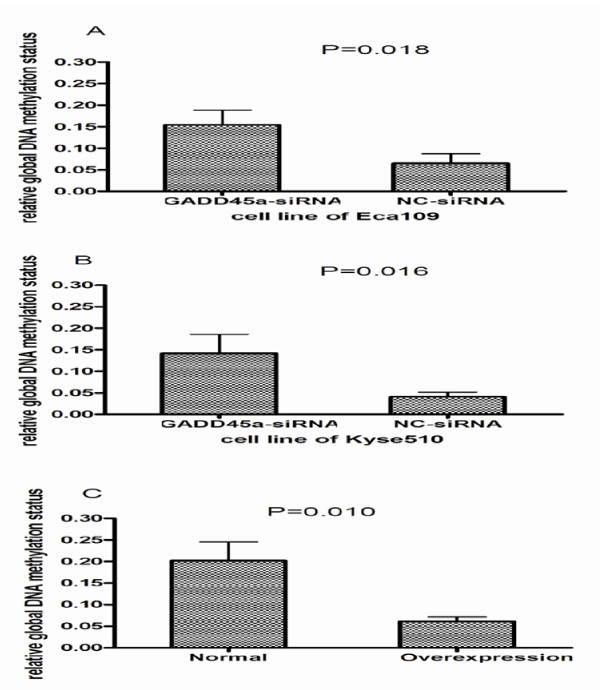
**A and B show that the DNA global methylation level in GADD45α-siRNA group was increased compared with NC-siRNA cells group**. C show that DNA global methylation level in over expression of GADD45α group was decreased compared with normal cells group.

The level of global DNA methylation was detected in the group of GADD45a-siRNA and NC-siRNA respectively. Then the result was that GADD45a-siRNA transfection increased global DNA methylation (Figure 6A and 6B).By making GADD45a overexpressed in normal human esophageal epithelial cells, it was found that the overexpression of GADD45a decreased global DNA methylation (Figure 6C).

## Conclusions

Overexpresssion and promoter hypomethylation of GADD45α gene and global DNA hypomethylation were found in ESCC tissues, which provide evidence that promoter hypomethylation may be the major mechanism for activating GADD45α gene in ESCC. The function of GADD45α in cell proliferation and apoptosis further demonstrated that overexpression of GADD45α contributes to the development of ESCC.

## Discussion

GADD45α, a nuclear protein, is implicated in the maintenance of genomic stability probably by controlling cell cycle G2-M checkpoint [18,23], induction of cell death [24], and DNA repair process [25-27]. It has been documented that GADD45α promotes gene activation by repair-mediated DNA demethylation[19]. As DNA repair gene, GADD45α is involved in the pathogenesis of many kinds of human cancers [28]. Recently, Zhang et al. reported that GADD45α play an essential role in gene-specific active DNA demethylation during adult stem cell differentiation [29]. But there is no report about expression and DNA methylation status of GADD45α gene and its role in ESCC. In this study, increased GADD45α expression was observed in esophageal squamous cancer tissues, and overexpression of GADD45α gene was associated with lymph node metastasis, and poor differentiation and TNM staging of ESCC. Hypomethylation in promoter of GADD45α and global DNA hypomethylation in tumor tissues of ESCC was also identified.

In our study, GADD45α mRNA and protein expressed higher in tumor tissue than in adjacent normal tissue, which may be due to DNA damage in epithelial cells induced by injury of esophageal squamous epithelium. When DNA damage takes place, GADD45α may act as a player in nucleotide excision repair [25,30]. Reinhardt, H. C et al. [31]found that following DNA damage, the p38/MK2 complex delocalized from nucleus to cytoplasm to stabilize GADD45α mRNA and MK2 phosphorylated PARN, blocking GADD45α mRNA degradation. Most DNA damaging agents and growth arrest signals (designated as non-IR treatments) have been found to induce GADD45α in cells regardless of p53 status [30]. GADD45α induction following DNA damage is rapid, transient and dose-dependent [32]. GADD45α induction by certain DNA damage-agents has been detected in a variety of mammalian cells. For example, rapid induction of GADD45α after MMS and UV treatments has been observed in every cell type tested to date. These cells include multiple mouse cell lines, human fibroblast, human lymphoblast and multiple human tumor lines [33,34]. Above all, GADD45α participated in DNA damage repair process; in return, DNA damage induced its overexpression.

DNA methylation is a major epigenetic mechanism for gene silencing and genome stability in many organisms [1,35,36]. In order to investigate the role of GADD45α in activating DNA demethylation, we explored the global DNA methylation condition and found global DNA hypomethylation in tumor tissues of ESCC. This finding was consistent with the published studies demonstrating incresed global DNA demethylation through GADD45α overexpression and DNA hypermethylation by scilencing GADD45α gene.[19]. Global DNA hypomethylation is considered as a feature of tumorigenic cells [37-39]; it can cause chromosomal instability, reactivation of transposable elements, and loss of imprinting [37,38,40]. In the experiment, we also found promoter hypomethylation of GADD45α in tumor tissues. Promoter hypomethylation has been hypothesized to lead to carcinogenesis by encouraging genomic instability [41]as well as by aberrant activation of oncogenes[42], thus promoter hypomethylation may participate in the development of ESCC.

We next investigated the biological effect of GADD45α in Eca109 and Kyse510 cell lines, and found that depletion of GADD45α by RNAi inhibited cell proliferation and promoted apoptosis. GADD45α play a role in the control of the cell cycle G2-M checkpoint. Takekawa et al. have reported that GADD45α interacts with MEKK4/MTK1 and activates the JNK/p38 signaling pathway that induces apoptosis and introduction of the GADD45α expression vector into tumor cells via transient transfection induces apoptosis [43]. GADD45α-mediated JNK/p38 activation is required for BRCA1-induced apoptosis [44] and UVB radiation-induced apoptosis is deficient in GADD45α-/- mouse epidermis [17]. In this study, our results showed that depletion of GADD45α by RNAi inhibited ESCC cells proliferation and promoted apoptosis, which suggested that GADD45α may be a novel and effective target for ESCC therapy.

Cisplatin (DDP) is the frequently-used chemotherapeutic agent shown to improve survival in patients with ESCC, as established by randomized controlled trials and therefore approved by the Food and Drug Administration for this use [45-48]. Resistance to chemotherapy, especially to DDP, has presented itself as a major obstacle in treatment of advanced ESCC. Many reports demonstrates that disruption of the apoptotic pathway seems to be a major mechanism of uncontrolled cell proliferation as well as resistance to chemotherapeutic agents[49]. Our finding showed that Eca109 and Kyse510 cells with knock-down GADD45α have decreased chemotherapeutic sensitivity to DDP, suggesting GADD45α may be play an important role in drug resistance of tumor cells. In next work, we will investigate the mechanisms that GADD45α decreases chemotherapeutic sensitivity to DDP.

In summary, overexpression and promoter hypomethylation of GADD45α gene and global DNA hypomethylation were found in ESCC tissues, which provide evidence that promoter hypomethylation may be the major mechanism for activating GADD45α gene in ESCC. The function of GADD45α in cell proliferation and apoptosis further demonstrated that overexpression of GADD45α contributes to the development of ESCC. However, the experiment of drug sensitivity indicated that GADD45α may be a protecting factor in DDP chemotherapy.

## Competing interests

The authors declare that they have no competing interests.

## Authors' contributions

BxW made all the experiment and wrote the manuscript. BlY and CC devised the experiment. BH, WxZ and YP made statistical data. MZ, ZkX and JqT made language amend of the manuscript. NY and XmZ checked and approved the manuscript. All authors read and approved the final manuscript.

## Authors' information

Bao xiang Wang: A medical Doctoral student in the second Xiang Ya hospital, majors in thoracic and cardiovascular surgery. He has worked for three years as a cardiovascular surgery doctor.
